# Facilitators and Barriers in Collaborations Between Community Health Workers with Primary and Well-Being Providers in Primary Healthcare in Belgium

**DOI:** 10.3390/healthcare12232348

**Published:** 2024-11-24

**Authors:** Hanne Apers, Caroline Masquillier

**Affiliations:** 1 Centre for Population, Family and Health, Department of Sociology, Faculty of Social Sciences, University of Antwerp, 2000 Antwerp, Belgium; 2 Primary and Interdisciplinary Care Antwerp Research Group (ELIZA), Centre for Research and Innovation in Care (CRIC), Faculty of Medicine and Health Sciences & Centre for Population, Family and Health, Faculty of Social Sciences, University of Antwerp, 2000 Antwerp, Belgium

**Keywords:** community health workers, primary healthcare, well-being providers, collaborations, facilitators, barriers

## Abstract

Background: Community health workers (CHWs) play a crucial role in bridging the gap between underserved populations and formal health. Collaborations between CHWs and health and well-being providers in primary healthcare are essential for improving access to and the quality of care for these communities. However, these partnerships require complementary strengths and specific conditions to succeed. This article addresses the limited knowledge on collaborations between CHW and primary health and well-being providers in Belgium’s CHW program. Methods: This study utilized a descriptive qualitative design. First, team collaboration data were gathered using a spreadsheet. Second, semi-structured interviews were conducted separately with 15 CHWs and 18 of their collaboration partners. The results were thematically analyzed. Results: CHWs collaborate with healthcare partners, partners with a social or societal focus, and government and educational institutions. The scope of collaborations spans sharing knowledge, connecting with the target group, and offering support to individual clients. Collaborations with healthcare providers tend to focus on individual referrals, with less reciprocity, while collaborations with other partners contribute more to outreach activities and addressing broader social determinants of health. Shared motivations and collaborative work methods facilitate collaboration, while internal organizational processes, lack of role clarity, and discrepancies can hinder successful collaboration. Conclusions: Strong local partnerships, well-defined roles, and mutual trust are essential for successful collaboration. The study findings highlight the importance of expanding collaborations to meet the diverse and intersecting needs of target groups. Effective program governance and policy are crucial in providing the flexibility necessary to address specific local requirements.

## 1. Introduction

Collaborations between community health workers (CHWs) and health and well-being providers play a crucial role in improving both access and quality of health care services [1,2]. The best opportunities for successful collaborations with health care providers arise when CHWs are integrated into primary health care [1]. CHWs complement primary health care by addressing the health needs of underserved communities, such as socioeconomically vulnerable populations [2,3]. Collaborations with CHWs contribute to reducing health inequalities within these groups and have beneficial effects on their treatment outcomes [1]. A key factor in the success of these collaborations is the complementarity of skills and knowledge between CHWs and regular primary health and well-being providers. On the one hand, CHWs enjoy a high level of trust in communities and have a sound knowledge of the relevant cultural, social, and economic contexts in which these communities live [4,5,6]. Through their close involvement and shared live experience, CHWs can act as a bridge to health care: their position within communities enables them to facilitate access to effective care, provide health information, and potentially coordinate preventive services [5,7]. On the other hand, regular health and well-being providers, such as (general) practitioners, nurses, social workers, and other care providers, have in-depth (medical) knowledge, specialized skills, and expertise that they can use to address complex medical and social problems and coordinate care [8,9].

Collaborations between CHWs and health and well-being providers offer significant benefits but are also accompanied by challenges that influence the quality and outcomes of these partnerships [10]. A successful collaboration between both parties, based on equality, respect, and mutual trust, in which effective communication plays a central role, is essential to achieve positive results [1,10]. Recent American research has shown that integrating CHWs into interprofessional teams can be a time-consuming process that sometimes lead to conflicts when traditional care models need to be revised [3]. This is often due to a lack of knowledge and understanding about the roles and added value of CHWs, which can lead to feelings of competition among regular healthcare providers. Research points to the importance of good coordination and division of responsibilities: both the roles of the CHWs and of the regular care providers must be clearly defined within the collaboration [1,10]. It is important that both parties are well informed about each other’s roles: clear communication or even training on this are crucial [1]. It is, therefore, essential to provide clear information about the contribution and capacities of CHWs, and regular healthcare providers should actively put effort in integrating CHWs into their work. Within a collaboration, a shared understanding of norms, values, and common goals among the different involved actors contributes to a smooth cooperation [1,10]. In addition, is crucial that the trust clients place in CHWs remains intact, as negative experiences in collaborations can undermine this trust [10].

Studies that have examined how CHWs are integrated into health care settings and teams emphasize the crucial importance of the social, economic, and political context in which this integration takes place [10]. These contexts vary depending on the country or region, so it is essential that policymakers take these contextual factors into account to enable the successful integration of CHWs into healthcare systems.

The Belgian CHW program, launched in 2021 by the Belgian Federal Minister of Public Health, aims to support individuals in socially vulnerable situations by helping them navigate the Belgian healthcare system [11]. Initially launched for one year to address health inequalities linked to the COVID-19 crisis, the program was extended for another year following a positive evaluation, and again for three more years, and is currently running until 2025. The National Institute for Health and Disability Insurance [RIZIV], which oversees health insurance and disability benefits, and the National Intermutualist College [NIC], a coordinating body for Belgium’s health insurance funds, were responsible for designing the program. The program operates under a federal governance structure with regional and local branches, and is currently implemented across ten cities in Belgium [12,13]. This governance structure creates a framework for collaboration at various levels, enabling the CHW program’s diverse objectives to be met. Locally, partnerships can focus on promoting individual health, while at the regional and federal levels, the emphasis shifts to enhancing and broadening Belgium’s overall health infrastructure through collaborations with various organizations and stakeholders. Given the varying local contexts in which CHWs operate, the focus of the local collaborations is tailored to the specific needs of each city, and allows for flexible responses to local demands. CHWs actively reach out to people in their own living environments, and support them to take more control over their health. Through establishing connections with local health and well-being providers, CHWs enhance the navigation and referral process for individuals.

To the best of our knowledge, little research has been conducted on collaborations between CHWs and health and well-being providers in primary healthcare in Belgium or even Europe. This study aims to help bridge this gap by examining the experiences of CHWs and their collaboration partners within a federal CHW program in Belgium. It investigates the nature of local collaborations, focusing on who CHWs collaborate with, how these collaborations unfold, key factors contributing to their success, and potential barriers that may impede them.

## 2. Methods

### 2.1. Study Design

The study applied a descriptive qualitative approach. To explore the nature of these collaborations, in-depth, individual interviews were conducted with a selection of both CHWs and their collaboration partners. By interviewing both the CHW and the partner separately, both perspectives on the collaboration were captured and unbiased responses were ensured. At the team level, open-ended questions were presented in a written format. Collaboration partners were defined as primary health and well-being providers in Belgian primary healthcare, or representatives of organizations focused on facilitating access to primary health or well-being care.

### 2.2. Participant Selection

The authors carried out a purposive selection of CHW respondents from all cities where the project is active, using socio-demographic data to ensure a balanced representation across regions, gender, migration and educational background, and age. Selected CHWs were contacted via email and invited to participate in the study. Before their interviews, CHWs were asked to complete a preparatory form listing all their individual collaborations with health and social primary care workers, both active or attempted collaborations. Using the information provided in these forms and through consultations with the CHWs, a purposive selection of partners were invited to participate in an interview.

The goal of the study was to achieve a well-rounded representation of collaborations, with a focus on primary health and well-being providers. Interviews were conducted with both partners who actively collaborated with CHWs and those with whom CHWs had attempted to establish partnerships that either did not materialize or were discontinued. This approach allowed for a comprehensive understanding of the dynamics and challenges associated with both successful and attempted collaborations.

In some cities, two types of partners were interviewed: a general practitioner (GP) and a representative from the social sector, as part of an additional study included in a master’s thesis in general practice.

### 2.3. Data Collection

Interviews were conducted at times and locations convenient to respondents during May and June 2023. Both CHWs and partners were questioned on various aspects of their collaboration, including how the partnership was initiated, factors contributing to its success, challenges encountered, and the evaluation of the collaboration.

In total, 15 interviews with CHWs and 18 interviews with partners were conducted. The interviews were conducted in Dutch or French, depending on the language preference of the participant. One interview was conducted in English. The interviews lasted between 1 and 1.5 h, and participants were given a small gift at the end of the interview as a token of appreciation. Each interview was recorded with an audio recorder, for which the participants were asked for their explicit consent during an informed consent procedure, which took place at the start of the interview. Data saturation was reached after the majority of interviews, but all scheduled interviews were completed to validate the findings.

The fillable spreadsheet that teams were asked to fill out had two tabs. The first tab collected descriptive information on collaborations, such as the number of collaborations, the type of partners, the level of collaboration, its duration, the frequency of contact, etc. The second tab featured open-ended questions aimed at gathering qualitative insights on a team level. These questions explored various aspects of the collaboration, including its initiation, factors contributing to success, obstacles encountered, and other relevant information.

### 2.4. Data Analysis

The audio recordings were transcribed verbatim by a certified organization under a data processing agreement to ensure the protection of the information. The interview transcripts were imported into the qualitative analysis program NVivo 14. These were analyzed according to the principles of thematic analysis [14,15]. This methodology consists of six steps, which were applied in a flexible manner: (1) familiarizing with the data by reading and rereading the interview transcripts, (2) generating initial codes that emerge from the data and mapping the content of the interviews, (3–5) searching, analyzing, and defining themes that reflect connections and explanations from the initial codes, and (6) processing the analysis results in a research report. 

The first author generated initial codes and subcodes from the data, including categories like ‘role clarity’ (with subcodes such as ‘lack of role clarity’), ‘communication strategies’, and ‘trust’. In the second step, these codes were reorganized according to the major themes aligned with the research questions and objectives, such as ‘facilitators’, ‘barriers’, and ‘collaboration process’ and minor subthemes. An iterative process followed, during which both authors reviewed and discussed the preliminary coding results, refining the codebook and addressing any inconsistencies. The interview data were then triangulated with the spreadsheet data, leading to the categorization of partner types and collaboration forms outlined in the results. The subtitles in the results section correspond to the major themes identified during analysis, while the subsections represent the most significant codes and subthemes within those themes.

### 2.5. Ethical Approval

The study was ethically approved by the independent Ethics Committee for the Social Sciences and Humanities (EASHW), installed by the Executive Board of the University of Antwerp (SHW_2023_7_1). At the start of each interview, participants were given explanations about the research procedure, and their informed consent was obtained. After the pseudonymized transcription of the recordings, these recordings were removed and deleted.

## 3. Results

The results highlight a broad spectrum of collaborations within the Belgian CHW program. The first part of the results outlines the different types of partners and collaborations. The second part highlights the factors that facilitate or hinder their success.

### 3.1. Types of Collaborative Partners

The types of partners that Belgian CHWs collaborate with can be grouped into three main categories: healthcare partners; partners with a social or societal focus; and government and educational institutions. Table 1 provides an overview of these categories, including their respective subcategories and examples of partners within each.

Several observations can be made regarding the data. Contrary to expectations, there is little or no collaboration with pharmacies and individual GPs, except for those affiliated with community health centers. Most partnerships involve a wide range of local services and organizations, categorized under ‘social or societal actors’, each with unique goals and missions. The specific organizations involved vary by local context, but common themes include health and well-being, psychosocial support, poverty assistance, education and training, social justice, youth work, and housing.

In collaborations with non-individual partners—such as organizations, institutions, or health centers—CHWs often work with designated contact persons, such as social workers managing client cases (e.g., OCMW social workers), resulting in different contacts per case. When collaborating with healthcare institutions, CHWs primarily interact with social workers or community workers, rather than medical staff. In schools, CHWs engage mostly with institutions that serve their target group, such as schools offering language courses to newly arrived migrants.

### 3.2. Types of Collaboration

A collaboration between a CHW and a local partner can take various forms and typically develops organically. The nature of the collaboration often depends on the specific context and goals of the partnership. Table 2 outlines a categorization of these collaboration types, with three main categories: collaborations aimed at sharing expertise and knowledge, those aimed at reaching the target group, and collaborations to support an individual client.

Our research pointed out that most collaborations focus on supporting individual clients. These collaborations can be either one-way—such as a CHW referring a client to a general practitioner or consulting a health insurance employee for administrative support—or interactive, where the CHW and partner regularly exchange services. In some cases, collaborations involve a more integrated approach, for instance when a CHW and a mutuality employee work intensively together to apply for disability benefits for a client. Notably, within the context of (primary) healthcare, collaborations predominantly involve one-way referrals and introductions by the CHW, with medical care providers relying less frequently on the services of the CHW.

*“Collaborations with care providers… I cannot tell you that these are collaborations, I would say they are orientations. Personally, I had a meeting with the medical center [in the district where the CHW works], it was really very open. They refer people to us, but it is not a collaboration […] The work we do with care providers is that we make contact.”*

(CHW, Brussels)

Equal collaborations between CHWs and medical care providers are rare. However, such equal partnerships are more common with health insurance organizations (which are partners within the CHW program), social or societal focus partners, and educational and government institutions. Since these organizations typically address a broader range of issues related to health, the collaborations often focus on addressing social determinants of health, such as poverty or inadequate housing.

Collaborations focused on reaching the target group, such as joint outreach efforts and organizing activities or drop-in sessions, are more commonly seen with organizations that have a social or societal focus or with educational institutions. This type of collaboration is less frequent with healthcare services. While some CHWs have established strong relationships with neighborhood health centers, and occasionally organize activities or drop-in sessions with them, these instances are less common and often depend on the willingness of the specific health center.

Collaborations are dynamic and can evolve over time, shifting from one form to another or exhibiting multiple forms with the same partner. For instance, a one-time referral to a non-profit organization might develop into an ongoing outreach partnership. Conversely, a regular collaboration with an individual care provider may be gradually phased out if, for example, differing visions emerge.

The local context and the policy environment of the city or district where the CHW program operates significantly influence the development of collaborations. In areas where a district or neighborhood program was already in place, or where a policy officer supported the program’s integration, CHWs were able to establish collaborations more easily. Announcing the program to neighborhood organizations and care providers went smoother, leading to quicker collaborations. For example, CHWs could often join existing neighborhood meetings or organize walk-in sessions with other organizations. In some districts, a coordinator facilitated connections between CHWs and local organizations.

### 3.3. Facilitators of Collaborating

Collaboration between CHWs and primary health and well-being partners is facilitated by both their shared motivations and collaborative methods. When both parties have aligned goals and compatible working styles, it enhances the initiation and progression of their partnership.

#### 3.3.1. Shared Motivations

The outcomes of the collaboration motivate both parties to work together: it significantly enhances the impact of their efforts. One key outcome is that collaboration improves the reach and effectiveness of targeting people in vulnerable situations. Partnering with organizations that already serve the same target group allows CHWs to connect with their audience more efficiently and extensively, often through joint activities or outreach events. Through their collaboration with CHWs, some partners can reach individuals they previously could not reach, benefiting from the CHW’s ability to build trust with clients who may be skeptical of traditional healthcare.

“*I am currently going through a process with someone who is somewhat vulnerable mentally, who could not come to me herself. The community health worker came with her. She does not answer to calls, so I could not actually reach that lady to follow up. That has always been facilitated through the community health worker. Now we have reached the point where the lady comes to me independently, because she knows me and if I send a text message first and then call, she does answer. So if that community health worker did not help support that process, that lady would never have come to us.*”

(Mutuality employee)

A second key outcome of collaborating is the enhanced support and follow-up of individual clients. By working together and sharing information, CHWs, care providers, and organizations can more effectively monitor clients’ health and well-being. This collaborative approach enables quicker identification and resolution of issues, leading to improved treatment outcomes. It allows CHWs to refer clients more efficiently and guide them toward the appropriate services. For instance, several CHWs reported that their role often facilitates faster appointment scheduling and access to care for individual clients. Collaboration partners noted that the CHW’s approachable way of working is highly valuable for client follow-up and guidance. By using a supportive and flexible approach, CHWs bridge cultural and language barriers and offer services to help the client navigate the health system. Furthermore, CHWs can offer a reliable point of contact by being present at fixed drop-in times at partner organizations. A key success factor is the program’s dynamic approach and its ability to adapt to the specific needs of the local context. Collaboration progresses smoothly due to strong local networks CHWs have with well-established health and welfare organizations.

Another shared motivation for collaboration is the complementarity of strengths and expertise between both CHWs and their partners. By working together, organizations can contribute with their specialized knowledge in areas relevant to the CHW, while CHWs can offer their expertise to organizations that may lack healthcare knowledge. For instance, in self-organizations with limited healthcare involvement, CHWs can play a crucial role by helping facilitate access to health information and services. Additionally, CHWs often serve as a bridge between different organizations, helping to consolidate expertise and build a network of knowledge and resources.

#### 3.3.2. Collaborative Methods

In collaborations between CHWs and their collaboration partners, the methods and dynamics of their interaction are essential to the success of their partnership. Both CHWs and their partners emphasized the importance of trust in fostering a productive collaboration. Trust plays a fundamental role, as it establishes an environment in which the collaboration partners can rely on each other, can share concerns, and solve disagreements constructively. Participants argued that clear and open communication is equally essential, as it fosters better cooperation by ensuring a transparent exchange of information. Additionally, collaboration partners highlighted the quality of the CHW’s work, noting their reliability, punctuality, professionalism, and expertise as significant facilitators for collaboration.

A flexible attitude and alignment in vision between the CHW and their partners also enhance the effectiveness of the collaboration. Shared goals and values promote smoother interactions, particularly when both parties are working towards the same objective. CHWs specifically highlighted the importance of qualities such as empathy, compassion, and the ability to understand the individual’s circumstances. In individual client guidance, keeping the person at the center, with both parties sharing the common goal of ensuring the person’s well-being, supports a respectful and open collaboration.

“*This is the social field. So I believe it is very important that everyone in the social field understands this. It is not a business need.... Humanity is what we are working on […] Because it is all for the well-being. […]*”

(CHW Brussels)

### 3.4. Barriers in Collaborating

The research identified several barriers to effective collaboration. These barriers often stem from practical issues, particularly during the initial stages of the CHW program, when various organizational challenges still needed to be addressed. Other barriers arose from unclear roles for the CHWs and a limited understanding of each partner’s operations. Additionally, discrepancies in the content and methods of collaboration further contributed to these obstacles.

#### 3.4.1. Internal Organizational Processes

Several [potential] collaboration partners were reluctant to work together at the start of the program. This reluctance was partly due to the CHW’s busy schedules and high workloads from the COVID-19 response, which made it difficult to engage effectively with them. Also the program’s initially announced one-year duration, led some partners to question the feasibility of achieving meaningful outcomes within such a short time frame. For some, the program appeared to be yet another temporary initiative in a series of projects, diminishing their commitment to engage. The rapid and at times disorganized start-up in the first year further contributed to an unreliable perception of the program. The absence of a fixed workplace or official status within the healthcare system for CHWs also created a perception of unprofessionalism, further discouraging collaboration.

*It (the CHW-program) was suddenly there, ‘poof’, a kind of meteoroid, or something […] Well, they (=CHW) also indicate that they do not have an office, that they do not have a workplace, that they are actually placed everywhere and nowhere, which also creates uncertainty, because in the community health centers, or other places where they are, they are also seen as outsiders. […] What are they doing here, and what can we tell and not tell these people, and what is their task, what is our task?*

(Individual well-being caregiver)

The internal processes of partner organizations can also complicate collaboration. Structural shortages among general practitioners, dentists, and specialists, such as psychologists and psychiatrists, have had a significant impact on collaboration efforts. Many of these care providers operate with patient stops or long waiting lists, making active collaboration challenging, despite potential interest from these professionals. While their interest in the CHW program can sometimes help to facilitate access for a CHW client, CHWs frequently need to find alternative providers with the availability to take on new patients. In larger organizations, frequent staff turnover can mean that new employees must repeatedly familiarize themselves with the CHW and the program, or that client files need to be transferred multiple times, complicating continuity of care. It is also important for collaborators to have aligned attitudes and visions. If their perspectives do not match or if they struggle to reconcile differences, it can hinder effective collaboration.

#### 3.4.2. Lack of Role Clarity

A frequently cited challenge was the lack of clear role delineation for CHWs. To start, the term ‘Community Health Worker’ was not widely recognized, limiting partners’ understanding and familiarity. The roles and responsibilities of CHWs often remained unclear. While most collaboration partners had a general understanding of what CHWs do, this knowledge was often vague or incomplete, leading to confusion or frustration on both sides. For instance, collaboration partners might seek advice from CHWs on issues beyond their designated roles, such as questions about employment or housing.

*“I got a question like: do you want to come along to ask for a reduction in the rent? I said: “No, I can’t do that.” And that person was angry, I could see it on his face, he was not happy. I say: “Sorry, but that is official, I can’t do that.”*

(CHW Flanders)

Moreover, the absence of clear role boundaries sometimes led to CHWs being perceived as additional staff, resulting in requests for them to take on administrative tasks for the partners. Some potential partners did not perceive the CHW as professional care providers. Furthermore, the diverse personalities and working styles of different CHWs could add to the confusion among collaboration partners about the scope of CHW duties. A significant challenge was the unclear demarcation of roles concerning translation and interpretation. Although collaboration partners often need assistance in these areas, acting solely as an interpreter falls outside the designated responsibilities of CHWs. This discrepancy frequently led to role conflicts for CHWs, who face a difficult choice: refusing to interpret might result in suboptimal care for the client, while agreeing to interpret can inadvertently create the expectation that this task is part of their role.

In some instances, the situation reversed, with CHWs lacking a thorough understanding of how their partners operated. This gap in knowledge sometimes led to inappropriate referrals or assessments. For example, when a CHW referred a client to a psychologist without fully considering the client’s cultural background and understanding of mental health care, the psychologist was unable to provide effective support. This situation resulted in the partner feeling that the referral could have been better managed if the CHW had been more informed about the nature of the care provided. In some instances, collaboration was obstructed due to differing perspectives on the client’s best interests. When a CHW then adopted a protective stance towards clients, they sometimes challenged the partner’s methods or interfered in external collaborations, which could damage the working relationship and undermine trust.

#### 3.4.3. Discrepancies in Collaboration

In certain neighborhoods, cities, or regions, the overlap with other organizations or projects offering similar services created confusion among potential partners. CHWs sometimes felt that these overlapping organizations viewed them as “competition,” especially when there were similarities in tasks or objectives, or due to financial interests. Although partners did not explicitly use the term “competition,” it was evident that some questioned the added value of CHWs in their work. However, some of these partners had limited understanding of the role of the CHWs.

In some cases, discriminatory or racist attitudes of health providers were reported by CHWs, where for instance, services were refused to CHW clients, such as individuals without legal residency. CHWs terminated or refused to establish collaborations in those instances.

## 4. Discussion and Conclusions

Collaboration between CHWs and primary care is crucial for enhancing both access to and the quality of healthcare services for underserved populations [1,2]. This study explored the nature of CHW collaborations with primary care partners within the Belgian context. The findings identify key prerequisites for effective collaboration and highlight a gap in the literature regarding the need for diverse collaboration approaches to address the social determinants of health. This section concludes with recommendations for strengthening collaborations and integration into primary care, along with a discussion of the study’s limitations and suggestions for future research.

### 4.1. Collaboration as Key to CHW Integration in Primary Care

CHWs occupy a unique position bridging the gap between the communities they serve and the formal health system. This positioning offers substantial advantages for their work but also necessitates close integration into the health system [10,16,17,18]. Health systems are often complex and dynamic, characterized by a range of actors with varying interests, perspectives, and values [19,20]. Integrating CHWs into these systems presents a continual challenge and requires a delicate balance among different stakeholders [10]. Recent research in the scientific literature highlights a shift towards a more horizontal and integrated approach, recognizing CHWs as a crucial and sustainable component of health systems [5,17,21]. There is growing acknowledgment of the potential for CHWs to take on broader responsibilities within integrated primary health care [1,17,19,22]. Effective integration of CHW programs into primary healthcare systems can enhance the sustainability and credibility of these programs, clarify CHW roles, and foster collaboration with higher-level health system actors [23]. This integration allows CHWs to enhance the visibility of existing health services and provide accurate information about primary care [24]. Furthermore, CHWs can represent the communities they serve within healthcare systems [1]. Conversely, insufficient integration of CHW programs can lead to fragmented service delivery, limiting their effectiveness [25,26]. Establishing effective collaborations with care providers is, therefore, crucial, as it involves coordinating the diverse expectations and needs of both community and care sector actors [25]. A flexible approach is essential to facilitate engagement with all relevant parties and develop potential collaborations.

#### CHW Collaborations in Belgium

In recent years, the Belgian CHW program has increasingly focused on establishing effective collaborations [13]. From its start, the program has emphasized the importance of local adaptation and integration: CHWs are encouraged to adapt their efforts to the local context and form specific partnerships tailored to each neighborhood [12]. The importance of responsiveness to the local context for the success of CHW programs is also highlighted in the international scientific literature [19,21,27]. Tailoring CHW activities to the local context allows them to address local needs effectively and ensures that the program remains adaptable to changing neighborhood circumstances [16,19]. This approach is viewed as more sustainable and is associated with improved health outcomes over the medium to long term [16]. CHWs and collaboration partners working in neighborhoods with established networks, such as those facilitated by neighborhood or district managers, found that setting up collaborations was relatively easy and swift, consistent with findings in the international literature [28].

As with CHW programs globally, the Belgian CHW program has established partnerships with a diverse array of stakeholders [25]. Based on the research results, we categorized these collaborations into three main groups: healthcare actors, social or societal organizations, and government and educational institutions. These partnerships mirror those described in the scientific literature, involving collaborations with government agencies, civil society organizations, primary care providers, schools, and community representatives [25]. The nature and depth of these collaborations vary widely, reflecting the findings of international studies [1,25]. In this study, we categorized collaborations based on their primary objectives: sharing expertise and knowledge, reaching the target population, or providing individual support.

### 4.2. Establishing a Good Collaboration

Clear and supportive collaborations between CHWs and their partners offer numerous advantages. Such collaborations play a pivotal role in enhancing access to care and improving the overall quality of health systems [29]. Effective partnerships can also help legitimize the role of CHWs, both within healthcare systems and in their interactions with target populations [10]. Study participants underscored the vital importance of collaboration, emphasizing its contribution to more effective outreach to target groups. They reported that collaboration improves follow-up and referral processes, as both parties can leverage complementary expertise and knowledge. Moreover, these collaborative efforts allow individuals with specific needs to connect with CHWs more efficiently. These findings are consistent with international research [1,4,5].

Establishing successful collaborations is a gradual learning process that requires time, careful planning, and coordination [3,30]. A key element identified by the interviewees is the development of trust between CHWs and their collaboration partners, which is essential for fostering effective partnerships. This aligns with the international literature that emphasizes the role of respect and mutual trust in achieving positive outcomes [1,10]. Another important factor highlighted by the participants is clear and open communication. Indeed, effective communication between healthcare providers and CHWs, along with the use of protocols and referral letters, can significantly strengthen the connection between CHWs and health facilities [10,31]. Additionally, recognition of the CHW role, combined with a shared understanding of norms, values, and common goals, further contributes to seamless collaboration.

Interviewees also highlighted potential obstacles to successful collaboration. CHWs often reported that they sometimes felt to be not treated as equals by their collaboration partners, particularly when assigned primarily administrative tasks. This is a common issue globally, where CHWs are sometimes not regarded as integral members of healthcare delivery teams, which can negatively impact their work [10,32]. Conflicts can arise when CHWs are seen as competitors rather than collaborators [3]. Time pressures and heavy workloads of healthcare providers were also cited as barriers to initiating or maintaining collaborations. Indeed, external factors, such as time constraints and the willingness or “goodwill” of primary care actors, can influence collaboration efforts [3]. Additionally, collaboration partners emphasized that a chaotic program start and an initially short program duration hindered effective partnerships.

A key aspect of successful collaboration, highlighted by multiple interviewees, is a clear role and task division. A well-defined and widely understood description of the CHW’s role, supported by all involved actors, is indeed essential for fostering collaboration and ensuring the CHW’s contributions are recognized and valued [10,28,33]. Both the roles of CHWs and regular healthcare providers must be distinctly defined within the collaboration [1,10]. The likelihood of success is higher when all healthcare actors are well informed about the specific role and responsibilities of CHW [34]. A clear role description helps align the expectations of both healthcare providers and CHWs [30], and facilitates the integration of CHWs into the healthcare system [10,19]. Role ambiguity can create problems, potentially undermining the credibility and effectiveness of CHWs [10,31,33]. It is essential to strike a balance where CHWs are recognized as valuable contributors, rather than being viewed as cheap labor [35]. Furthermore, as illustrated in the study results, when healthcare providers see the value of the work of the CHW, they are more likely to support the activities of the CHW [5].

### 4.3. Diverse Collaborations to Tackle Social Determinants of Health

The study highlights a gap in the literature by revealing that certain types of collaborations are more prevalent with specific types of partners. This differentiation suggests that the nature of the collaboration is often shaped by the professional orientation of the partner, with healthcare actors focusing on clinical support and social actors playing a larger role in community engagement. In the context of the Belgian CHW program, collaborations that focus on individual support are more commonly established with healthcare actors. These partnerships primarily center on individual follow-up, where CHWs typically refer or guide individuals to healthcare providers, resulting in largely one-sided interactions. Reciprocal exchanges are less frequent, and broader forms of collaboration are rare. When such interactions do occur, they are more likely to involve socially oriented healthcare professionals, such as social workers in hospitals or receptionists in community health centers. In contrast, collaborations aimed at reaching the target population, which include activities such as information dissemination, are more frequently established with partners who have a social or societal focus.

The more intensive collaboration with social actors in the CHW program is largely driven by the complex and multifaceted nature of the issues CHWs encounter, which often require cross-domain collaboration. The health of target groups within the CHW program is frequently shaped by social determinants, such as housing and employment, that extend beyond the healthcare system [10,11,13]. As a result, CHWs must engage with a broader network of partners to address these intersecting needs. To ensure the program’s success, it is crucial to maintain a dual focus: promoting effective collaborations within the healthcare system while also addressing the wider social and economic challenges faced by target groups [10,34]. Research shows that a mismatch between community needs and the services provided by CHWs can result in dissatisfaction among target groups and feelings of frustration and powerlessness among CHWs [5]. Therefore, it is essential to focus on developing partnerships that encompass both health and social domains to ensure a comprehensive approach to community health.

### 4.4. Recommendations for Policy and Practice

This research highlights several key recommendations to optimize future collaborations within the CHW program. To ensure successful implementation of partnerships in practice, it is essential to engage in a participatory decision-making process that includes input from CHWs and their team responsible, aligning the program with the needs and expectations of the field [36]. Through a thorough context analysis, before geographical expansion, the unique contributions of the CHW program to the local field can be identified [28]. Before the start of a partnership, a clear communication of the program’s mission and vision should be established, in which the roles of the CHW are well defined [5]. Additionally, conducting yearly evaluations can enhance the collaboration effectiveness [37].

At the policy level, the federal and regional governance structures of the Belgian CHW program are essential for promoting effective and sustainable partnerships. By facilitating connections at the federal or regional level with other organizations and initiatives, these governance structures can act as catalysts for creating partnerships between local teams. However, the development of these structural relationships is not solely the responsibility of CHW programs; policymakers also have a critical role to play. Integrating CHWs into the broader policy vision as an integral part of the health system is essential for creating an environment that supports effective collaborations [25]. Since CHW programs are shaped by the policy framework in which they operate, this framework significantly influences the establishment and success of collaborations [16].

### 4.5. Research Limitations

Although this research provided valuable insights, it also has several limitations that should be taken into account when interpreting the results. First, our data collection primarily focused on collaborations within the local context, and does not provide an understanding of collaborations at the broader policy level. The findings highlight significant variations influenced by the local context, as well as the important impact of higher-level policies. Future research in this area could benefit from a comparative analysis across different local contexts, and incorporating the role of those broader policy levels. Second, the selection of interview participants limits the generalizability of the findings as in each city, only one CHW and one collaboration partner were interviewed. However, this selection was designed to ensure that, given the available resources, all cities where CHWs were active were involved in the study, allowing for the identification of common dynamics across locations. We chose not to include a demographic description of participants to protect their privacy. CHW participants were purposively selected for balanced representation, and collaboration partners were chosen to reflect a diverse range of primary and well-being providers, as well as both successful and challenging collaborations, to provide in-depth insight. Third, selection bias during participant selection cannot be excluded as we asked coaches and CHWs to provide contact details for those willing to participate. Interviews were conducted only with individuals who agreed to participate, which may distort the overall picture. Data from collaboration partners were also collected through coaches and CHWs, potentially impacting the representativeness of the sample. To mitigate an overly positive representation, we aimed to capture both successful and challenging collaborations, although this bias cannot be entirely eliminated. Lastly, the qualitative nature of the research is exploratory, meaning that the findings may not be generalizable to all collaborations within CHW programs. Instead, they provide in-depth insights into the local dynamics of collaborations within the Belgian CHW program. Future research could benefit from a mixed-methods approach, integrating quantitative data to validate and expand upon these qualitative insights.

## Figures and Tables

**Table 1:** Types of partners.

Type Partner	Examples
**Healthcare partners**	
Individual care providers	Individual general practitioners, dentists, specialists
Healthcare institutions	Hospitals, specialized centers
Healthcare services	Neighborhood community health centers and medical homes, mobile teams, home care services
Mutualities (health insurance organizations)	Christian Mutality, Solidaris, Liberal Mutuality, Helan, Neutral Mutuality
**Partners with a social or societal focus**	
Local organizations or individual actors with a societal focus and volunteer organizations	Local neighborhood or district services and organizations, non-profit associations (NGOs), homeless shelters, food distribution, legal services, etc.
Welfare organizations	Centers for General Welfare, service centers
Religious institutions	Mosques, Catholic churches, etc.
**Government and educational institutions**	
Schools	Dutch as a second language (NT2) schools, basic education
Public institutions	Public Centre for Social Welfare (OCMW), integration services, city services, Flemish Employment Service (VDAB)

**Table 2:** Collaboration types.

Type of Collaboration	Explanation
No active collaboration	CHW and organization/care provider/are aware of each other’s existence but do not collaborate
**Collaborations aimed at sharing expertise and knowledge**	
Sharing expertise or knowledge	CHW and organization/care provider/consult each other for information, knowledge, and expertise on a specific topic
**Collaborations aimed at reaching the target group**	
Drop-in moments/workplace	The CHW/local CHW team uses the spaces of a collaboration partner as a workplace or holds drop-in moments during the services of the partners to meet clients
Outreach work	CHW and collaboration partner have the same target audience and join forces to reach this group, for example by going out on the streets together
Organization of activities	The CHW/local CHW team and collaboration partner organize activities together, such as information sessions on health themes
**Collaborations to support an individual client**	
Orientations	The CHW refers a client to a partner, or vice versa
Referrals	The CHW directs the client to a partner, i.e., the CHW accompanies the client to the partner one or more times, with the intention that the client can then establish contact independently
Guidance	The CHW and the partner guide a client together in a trajectory

## Data Availability

Data are available upon reasonable request from the authors.
